# Development and validation of a new instrument to measure perceived risks associated with the use of tobacco and nicotine-containing products

**DOI:** 10.1186/s12955-018-0997-5

**Published:** 2018-09-21

**Authors:** Stefan Cano, Christelle Chrea, Thomas Salzberger, Thomas Alfieri, Gerard Emilien, Nelly Mainy, Antonio Ramazzotti, Frank Lüdicke, Rolf Weitkunat

**Affiliations:** 1 Modus Outcomes, Spirella Building, Letchworth Garden City, SG6 4ET UK; 2 Philip Morris Products S.A, Quai Jeanrenaud 3, 2000 Neuchâtel, Switzerland; 3 Institute for Statistics and Mathematics, Institute for Marketing Management, University of Economics and Business (WU Wien), Welthandelsplatz 1, 1020 Vienna, Austria; 4 Covance Market Access Inc, 10300 Campus Point Drive, Suite 225, San Diego, CA 92121-1511 USA; 5 Philip Morris International Management S.A, Avenue de Rhodanie 50, 1007 Lausanne, Switzerland

**Keywords:** Risk perception, Modified risk tobacco products, Psychometrics, Public health

## Abstract

**Background:**

Making tobacco products associated with lower risks available to smokers who would otherwise continue smoking is recognized as an important strategy towards addressing smoking-related harm. Predicting use behavior is an important major component of product risk assessment. In this context, risk perception is a possible factor driving tobacco product uptake and use. As prior to market launch real-world actual product use cannot be observed, assessing risk perception can provide predictive information. Considering the lack of suitable validated self-report instruments, the development of a new instrument was undertaken to quantify perceived risks of tobacco and nicotine-containing products by adult smokers, former smokers and never-smokers.

**Methods:**

Initial items were constructed based on a literature review, focus groups and expert opinion. Data for scale formation and assessment were obtained through two successive US-based web surveys (*n* = 2020 and 1640 completers, respectively). Psychometric evaluation was based on Rasch Measurement Theory and Classical Test Theory.

**Results:**

Psychometric evaluation supported the formation of an 18-item Perceived Health Risk scale and a 7-item Perceived Addiction Risk scale: item response option thresholds were ordered correctly for all items; item locations in each scale were spread out (coverage range 75–87%); scale reliability was supported by high person separation indices > 0.93, Cronbach’s alpha > 0.98 and Corrected Item-Total Correlations > 0.88; and no differential item functioning was present. Construct validity evaluations met expectations through inter-scale correlations and findings from known-group comparisons.

**Conclusions:**

The Perceived Risk Instrument is a psychometrically robust instrument applicable for general and personal risk perception measurement, for use in different types of products (including cigarettes, nicotine replacement therapy, potential Modified Risk Tobacco Products), and for different smoking status groups (i.e., current smokers with and without intention to quit, former smokers, never smokers).

## Background

Cigarette smoking causes many serious diseases [1]. The best way to reduce the adverse health consequences of smoking is to stop smoking [2]. For smokers who choose to continue to smoke, reducing exposure to toxicants and safer delivery of nicotine are among the strategies that have been introduced to reduce the risk of smoking-related diseases [3]. As new tobacco products, which may be a less harmful alternative to continued smoking, become increasingly available (e.g., smokeless tobacco, e-cigarettes, heat-not-burn products), this has created new challenges for policy makers [4]. In the United States (US), a regulatory framework has been put in place since 2012 for manufacturers to market a modified risk tobacco product (MRTP) – that is, any tobacco product that is sold or distributed for use to reduce the risk of tobacco-related disease associated with commercially marketed tobacco products [5]. 

As population health impact is a function of product risk and product uptake distribution, predicting product use prior to market launch is an important component of product risk assessment. In particular, the effect that an MRTP’s marketing will have on consumer understanding and perception is an important consideration as it is essential that the product communication materials be an accurate, non-misleading, and scientifically substantiated reflection of the product characteristics, permitting adult smokers to understand the risks and benefits compared to other tobacco products, without encouraging non-smokers to initiate or reinitiate tobacco use [6]. Part of validating these requirements is the assessment of consumer risk perception, as such perceptions might be crucial determinants of product use among both current tobacco users and non-users [7, 8]. At the same time, valid instruments to measure consumer responses to tobacco products are largely lacking [9] and there is currently no self-report instrument available that would allow the quantification of perceived risks of different tobacco and nicotine-containing products [10].

A self-report instrument should be: (1) appropriate to capture the individual perspective and include relevant and meaningful domains; (2) applicable across a wide range of tobacco and nicotine-containing products; (3) suitable for a range of respondent groups such as users and non-users; (4) underpinned by an appropriate psychometric measurement model; (5) straightforward to administer and score; and (6) applicable for clinical and population-based studies. These criteria reflect current standards of valid measurement in terms of qualitative aspects (i.e., relevant and meaningful domains as evidence of content validity) and quantitative requirements of construct validity (i.e., psychometric criteria), as well as regarding the practicability and usefulness. Other desirable psychometric properties include: unidimensionality (meaning that there is one underlying latent variable accounting for the observed item scores); separation of person and item parameter estimates (allowing for a detailed examination of the extent to which a set of items proposed to form a scale separates participants and allows for precise measurement); and lack of item bias with respect to subpopulations (thus the properties of the participants, their distribution and other characteristics, should not impact on the item properties). These psychometric properties support a metrological framework for the social sciences, and can be realized by using the Rasch model which: provides parameter separability, statistical sufficiency, and specific objectivity [11]; is embedded in Georg Rasch’s general philosophy of measurement [12]; and, is subsequently formalized in the language of measurement traceability [13] and uncertainty [14].

Previous research [10] showed the measurement of perceived risks typically relies on, at most, a small number of self-report items [9, 15–21]. However, single items, or short scales, do not allow for a comprehensive measurement of risk perception, and provide little insight into the underlying perceived risk continuum. Also, short scales tend to lack reliability, and, by implication, measurement precision [22]. And psychometric measurement models cannot be easily applied, limiting the quantitative assessment of construct validity. Current measurements of perceived risks are typically product-specific (e.g., for cigarettes) [15, 21, 23–25] or rely on a single statement of comparative risk between a pair of products [26, 27]. In the context of an MRTP assessment, flexible and indirect comparison is needed between all tobacco- or nicotine-containing products [5, 28]. Finally, existing approaches tend to focus on current users (e.g., cigarette smokers). Considering the lack of suitable validated self-report instruments, the objective of the present work was to develop a new instrument to quantify perceived risks of tobacco and nicotine-containing products by adult smokers, former smokers and never-smokers. Here, we describe the overall process and subsequently focus on the development of two scales addressing Perceived Health Risk and Perceived Addiction Risk.

## Methods

### Development of the draft instrument and pre-testing

To support the development of a conceptual framework and subsequent item generation, a literature review and a series of qualitative studies were conducted, including focus groups and expert opinion elicitation. All this qualitative research is described in detailed somewhere else [10], and is only briefly summarized below. Prior to formal psychometric evaluation, cognitive debriefing interviews (CDIs) and a pilot testing were conducted to ascertain the good comprehension and acceptability of the draft instrument.

#### Literature review

A systematic search of studies related to risk perception and tobacco products published between January 2000 and September 2012 was conducted in Embase® and MEDLINE®. Further sources were identified by three public health experts,^1^ covering quality of life research, consumer risk perception research, and scale development. A total of 136 papers were identified by database search, 36 by experts, leading to 42 papers being selected for a detailed review. The literature review revealed four broad domains with perceived health risk (including addiction risk) being the most widely captured domain (referenced in 24 papers). Less frequently, social, financial, and time-related aspects of perceived risk were addressed (referenced in 8, 2, and 2 papers, respectively).

#### Focus groups

In order to gain insights into the respondents’ perspectives, 29 focus groups were conducted in the US, UK, Italy, and Japan. Smoking status groups were defined in accordance with the World Health Organization (WHO) guidelines [29] and the Prochaska and DiClemente stages of change model [30] (see Table 1 for demographics). In the focus groups, conventional cigarette, electronic cigarette and a nicotine replacement therapy (NRT) samples were used to stimulate participants to discuss risks associated to tobacco and nicotine-containing products. The emerging domains showed wide overlap across countries. Health and addiction risks dominated the concepts in all countries except in Japan, where societal/social risks and material/financial risks were about equally prevalent.

**Table 1:** Participants in the Focus Groups, Cognitive Debriefing Interviews (CDIs) and Surveys

Variables	Focus Groups^a^	CDIs^b^	Survey 1 (*N* = 2020)	Survey 2 (*N* = 1640)
Sex, *n* (%)				
Male	109 (47.6)	42 (47.7)	932 (46.1)	792 (48.3)
Female	120 (52.4)	46 (52.3)	1088 (53.9)	848 (51.7)
Age (years), Mean ± SD	39.7 ± 12.7	–	45.0 ± 17.4	42.9 ± 16.3
18–25 years, *n* (%)	34 (14.8)	27 (30.7)	na	na
26–50 years, *n* (%)	136 (59.4)	34 (38.6)	na	na
51–65 years, *n* (%)	59 (25.8)	26 (29.5)	na	na
18–30 years, *n* (%)	na	na	560 (27.7)	509 (31.0)
31–45 years, *n* (%)	na	na	636 (31.5)	544 (33.2)
46+ years, *n* (%)	na	na	824 (40.8)	587 (35.8)
Race, *n* (%)				
Caucasian			1628 (80.6)	1309 (79.9)
African-American			152 (7.5)	128 (7.8)
Other			240 (11.9)	203 (12.4)
Education Level, *n* (%)				
High school and less	68 (29.7)	30 (34.1)	705 (34.9)	634 (38.7)
Some college and more	142 (62.0)	58 (65.9)	1315 (65.1)	1006 (61.3)
Other	19 (8.3)	–	–	–
Smoking Status, *n* (%)				
Adult smoker with no intention to quit	71 (31.0)	22 (25.0)	437 (21.6)	408 (24.9)
Adult smoker motivated to quit	39 (17.0)	22 (25.0)	461 (22.8)	408 (24.9)
Adult former smoker	62 (27.1)	22 (25.0)	516 (25.5)	407 (24.8)
Adult never smoker	57 (24.9)	22 (25.0)	606 (30.0)	417 (25.4)

*SD* standard deviation

^a^Nine focus groups, conducted in London (*n* = 3), Birmingham (*n* = 3), and Glasgow (*n* = 3); Four focus groups conducted in Rome and in Tokyo; Twelve focus groups, conducted in Atlanta (*n* = 4), Los Angeles (*n* = 4) and Philadelphia (*n* = 4)

^b^Forty CDIs conducted in London (*n* = 20), Manchester (*n* = 10), and Glasgow (*n* = 10); Forty eight CDIs conducted in Atlanta (*n* = 23) and Los Angeles (*n* = 25)

#### Expert opinion

Four experts^2^ in nicotine addiction, motivational aspects of consumer perception, and epidemiology proposed relevant themes and reviewed the identified domains. Expert opinions widely agreed with the findings of the literature review and the focus groups and facilitated the consolidation of all qualitative input to the conceptual framework. However, experts recommended health risk to others as a separate aspect of health risk, and addiction risk as a domain on its own. The final conceptual framework thus comprised five potential domains:

- Perceived Health Risk to Self. The perceived negative risk (or impact) of product use to the user’s physical health, ranging from minor immediate concrete manifestations of health risk (e.g., having poor gum health) to more serious long-term ones (e.g., having lung cancer);
- Perceived Addiction Risk. The perceived negative risk (or impact) that product use may have on the user’s sense of being addicted to using the product;
- Perceived Health Risk to Others. The perceived negative risk (or impact) to the physical health of nonsmokers when being around during product use (not to be confused with the category of general risk, i.e., the risk of active use of tobacco products for active users in general);
- Perceived Social Risk. The perceived negative risk (or impact) that product use will affect interpersonal interactions adversely or how the user is perceived by others;
- Perceived Practical Risk. The perceived negative risk (or impact) that product use may have on the user’s time and finances.

#### Item generation

Two versions of a sentence stem presented at the top of each page were generated for all items within a domain. For Perceived Health Risk to Self, one stem referred to the personal risk to the individual respondent (e.g., with regards to cigarette smoking: “What do you think is the risk, if any, to you personally of getting the following (sometime during your lifetime) because you smoke cigarettes …”). The other stem referred to the risk to a user of a product in general (“In general, what do you think is the risk, if any, to smokers of getting the following (sometime during their lifetime) because of smoking cigarettes …”). Similar sentence stems were used for other domains. The items themselves consisted of brief expressions mostly in the order of four to six words, e.g., “having mouth or throat cancer” or “having reduced stamina”.

 A five-point fully verbalized rating scale ranging from “*no risk”* to “*very high risk”* was used to allow for expressing a medium level of perceived risk (*“moderate risk”*). The option *“don’t know”* was added for respondents not relating to some items and therefore lacking a perception. Two English language versions of the new proposed Perceived Risk Instrument (PRI) were drafted for personal (PRI-P) and general risk assessment (PRI-G). The intention was that the PRI would be applicable to: (1) adult smokers with intention to quit, adult smokers with no intention to quit, adult former smokers, and adult never smokers; and to (2) tobacco and nicotine-containing products as well as Cessation (perceived risk from having smoked in the past).

#### Cognitive debriefing interviews

The draft versions of the PRI-P and PRI-G were presented to UK and US participants using the same sampling frame as for the focus groups (Table 1 for demographics). Overall, participants found the content to be comprehensive, the stems to be clear, and the items and response formats straightforward to complete. Importantly, participants could discriminate between the two versions of the PRI and to assess personal as well as general risks accordingly. A few minor changes were made to the draft version of the instrument, including: (1) the adjustment of stems for participants with different smoking status and for different product types; (2) the removal of two items in the health risk to others domain due to ambiguity and lack of relevance; and (3) improvements of the wording of some items.

In addition, feedback from never smokers suggested that it was challenging to assess their personal risk of products (specifically NRT) they would never consider using. This led to the decision not to administer the PRI-P to NRT and Cessation to never smokers. The final draft versions of the PRI comprised a total of 67 items each, related to five domains: *Perceived Health Risk to Self (31 items); Perceived Health Risk to Others (3 items); Perceived Addiction Risk (11 items); Perceived Social Risk (13 items); and Perceived Practical Risk (9 items)*.

#### Pilot field testing

After the qualitative stage, the five-domain draft PRI was administered in a pilot study (web-survey with 233 completers) to assess the feasibility of developing the five scales in parallel. Floor effects (between 12 and 41%) occurred for perceived social and perceived practical risks when applied to products other than cigarettes (CCs). It was concluded that developing scales for perceived social and perceived practical risk would in all likelihood, at this stage, not result in properly targeted scales with a broad coverage of the latent continuum. Rather, additional qualitative research appeared to be necessary. This led to the decision to, for the time being, solely focus on perceived health and addiction risks for the psychometric evaluation of the PRI. Perceived health and addiction risks also were the most widely addressed domains of risk in the extant literature [10]. Therefore, the quantitative field tests were restricted to the three health-related domains, i.e., Perceived Health Risk to Self, Perceived Health Risk to Others, and Perceived Addiction Risk

### Psychometric evaluation

For the psychometric evaluation of the draft PRI, two online cross-sectional surveys were conducted in the USA. Survey 1 served scale formation and item reduction, while Survey 2 was used for cross-validation of the PRI.

#### Design and procedure

Survey 1 and Survey 2 were designed as internet cross-sectional studies with stratified sampling of four subpopulations defined according to self-reported smoking status at the time of data collection. Respondents reporting having smoked at least 100 cigarettes in their lifetime and currently smoking at least one cigarette (no brand restrictions) per day (disregarding religious fasting) at the time of data collection were classified as adult current smokers. The latter were further divided into those with, and those without intention to quit, in accordance to Prochaska and DiClemente’s Stages of Change model [31]. Respondents reporting that they were former daily smokers and, at the time of study, had been quitting smoking more than 30 days ago, were classified as former smokers. Those who reported that they had never smoked at all, or who had never been daily smokers and had smoked less than 100 cigarettes in their lifetime, were classified as never smokers.

Within each smoking status group, quota sampling based on age, sex, and education was applied. A web-based data capture tool (i.e., *Confirmit Horizons* version 16) was used to gather responses from study participants from an opt-in proprietary database maintained by Toluna Group Ltd. (Wilton, Connecticut USA), consisting of individuals with expressed interest in participating in online survey research. The samples within each stratum were not fully representative in terms of exactly matching the structure of the US population. Rather, the sample composition served the purpose of scale development and satisfied the needs in this regard, such as adequate representation of each segment defined by the quota criteria.

In Survey 1, respondents completed the PRI for the assessment objects CC, the Tobacco Heating System (THS) 2.2 (a heat-not-burn Reduced Risk Product (RRP)^3^ developed by Philip Morris Products S.A.), a nicotine patch and Cessation (defined as having successfully stopped smoking and not using any tobacco and nicotine-containing product). In Survey 2, E-cigarettes were added to the assessment and nicotine patch was replaced by nicotine replacement therapy (NRT) as a general category. Participants were quota-randomized to pre-determined sequences so that an equal number of participants of each demographic stratum would be exposed to a specific sequence of product assessment. A minimum of 1600 completers, with an equal representation of each of the four subpopulations defined by smoking status, was estimated as an appropriate sample size for psychometric evaluation for each survey [22, 32, 33].

Survey 1 (administered between February and March 2014) and Survey 2 (administered between May and June 2014) were both approved by the New England Institutional Review Board and the participants received complete information about the study before agreeing with an informed consent form (ICF). The total participation time for each survey was between 30 and 45 min and participants were rewarded with 3500 points to exchange for vouchers or gifts at the reward partner network of the company hosting the survey (Toluna Group Ltd).

#### Measurements

Three draft scales each for the PRI-P and PRI-G were evaluated: Perceived Health Risk to Self (31 items), Perceived Health Risk to Others (3 items), and Perceived Addiction Risk (11 items). A 5-point response scale was used, with ratings ranging from 1 (no risk) to 5 (very high risk), additionally offering a “don’t know” option.

Tobacco use history was captured by the Smoking Questionnaire [34], addressing current and past use of tobacco-related products. Age, sex, education, income, and ethnicity were also captured (see Table 1).

In Survey 2, additional measures were administered for convergent validity assessment: (1) overall measures of the relative perceived risks associated to the five objects (i.e., CC, THS 2.2, E-cigarettes, NRTs, Cessation), based on two 100 mm visual analogue scales (VAS); one for overall health risk to self and one for overall addiction risk [16, 18]; and (2) five items addressing the participant’s perceived short and long-term consequences of smoking [35].

#### Data analysis

Survey 1 analyses aimed at identifying the items with the best psychometric properties. Perceived Health Risk to Self (31 items) and Perceived Health Risk to Others (3 items) were initially combined to explore the potential of forming one inclusive 34-item scale. The internal construct validity of the items was assessed by Rasch measurement theory (RMT) analysis, which examined: response scale ordering (presence of disordered thresholds which are indicative of inconsistent use of response options); scale targeting (percentage of coverage item threshold distribution), model fit (item and person fit statistics); local dependence (item residual correlations); reliability (person separation index); and differential item functioning (DIF) assessed by age, sex, education, smoking status as well as across different tobacco and nicotine-containing products, and across the application of the scales to personal risk and risk in general (see Table 2 for more details on the definitions and acceptability criteria for RMT analysis).

The application of the Rasch model was motivated by its useful properties such as parameter separation and raw score sufficiency [36]. Parameter separation ensures invariance as a consequence of specific objectivity [12] in the Rasch model. Specific objectivity means that item characteristics do not depend on the respondents who are instrumental in their estimation, and vice versa, respondent characteristics are independent of the items used in their estimation. Hence, comparisons of items, and respondents, are invariant [37]. In other words, the instrument works in the same way for all individuals [38]. Raw score sufficiency proves beneficial from a practical point of view as it permits a simple raw-score-to-measure conversion. At this stage, the unrestricted polytomous Rasch model, also known as the partial credit model, was used [37].

**Table 2:** Rasch Measurement Theory Analyses: Properties, Definitions and Acceptability Criteria

Property	Definitions and Acceptability Criteria
Targeting	Targeting refers to the extent to which the range of the target construct measured by each of the scales (i.e., perceived health risk and perceived addiction risk) matches the range of that target construct in the study sample. Better targeting equates to a greater ability to interpret the psychometric data with confidence [50]. This involves examination of the relative distributions of the item locations and the person measurements as well as of the plot of the person-item location distributions, showing the item locations and the person measurements on a common scale. There is no specific criterion. Essentially, the item locations should cover the sample adequately and the sample should cover the item locations adequately.
Fit	The items of the scales of the proposed instrument must work together (fit) as a conformable set, both conceptually and statistically. Otherwise, it is inappropriate to sum item responses to a total score and consider the total scale score as a measure of the target construct. When items do not work together (misfit) in this way, the validity of the scale is questionable [50]. The following statistical and graphical indicators of fit were investigated [51]: • Item discrimination: Fit residuals summarize the difference between observed and expected responses to an item across all respondents (item-person interaction). Fit residuals should ideally lie within ±2.5. Fit residuals lying outside this range imply misfit of the observed data to the Rasch model. Negative values indicate overdiscriminating and positive values underdiscriminating items. Due to the large sample size in Surveys 1 and 2 it was to be expected to find a substantial number of item misfits, but this indicator was still considered helpful as some items were expected fitting much worse than others. • Item fit: Chi-squared values summarize the difference between observed and expected responses to an item for groups (or ‘class intervals’) of individuals with relatively similar levels of ability (item-trait interaction). A chi-squared value with a low likelihood (*p*-value) implies that the discrepancy between the observed responses and the expected value is large relative to chance for that item. • Item response ordering: This involves the examination of the category probability curves (CPCs) and the threshold probability curves (TPCs) which show the ordering of the thresholds for each item. A threshold marks the location on the latent continuum where two adjacent response categories are equally likely. The ordering of the thresholds should reflect the intended order of the categories lower (‘no risk’) to higher (‘high risk’) values. Correct ordering supports the assumption that the response categories work as intended. Disordered thresholds indicate that the response categories for a particular item are not working as intended, and therefore that the scoring function for that item is not valid. • Local independence: This involves an examination of item residual correlations [52]. Correlations between the residuals should be low (< 0.30). In addition, residual correlations are assessed against the average of all residual correlations plus 0.3 [53, 54]. If residuals for item pairs are correlated > 0.30, this indicates that the response to one item depends on the response to the other item, i.e., the items are locally dependent [55].
Reliability	Reliability refers to the extent to which scale scores reflect random error [56]. This was assessed using the person separation index (PSI), which is an internal reliability statistic comparable to Cronbach’s alpha. The PSI quantifies the error associated with the measurements of individuals in the sample [56]. The PSI ranges from 0 (all error) to 1 (no error). A low PSI implies that scale items are not able to reliably separating individuals on the scale they define.
Stability	Comparability of PRI measures across different factors was based on tests of invariance (key criterion of successful measurement), implying that items mean the same to different participant groups under different conditions. This is assessed by means of a test for differential item functioning (DIF) [57]. Invariance was assessed according to demographic criteria (age, gender, education) as well as across different tobacco and nicotine-containing products, different subpopulations based on smoking status and across the application of the scales to perceived personal risk and perceived general risk. DIF is assessed by comparing observed residuals (i.e., the difference between expected responses under the assumption of no DIF and actually observed responses) across groups of participants defined by the DIF factor investigated (e.g., males versus females) and classified in several class intervals along the latent continuum measured by the scale.

Classical test theory (CTT) analyses were conducted on the item-reduced scales resulting from RMT analyses, including: assessment of data quality (proportion of missing data as an indication of a lack of acceptability); scaling assumptions (similarity of item means and variances, item-total correlations); scale-to-sample targeting (floor and ceiling effects, skewness of item scores); and internal consistency reliability (Cronbach’s alpha) (see Table 3 for more details on the definitions and acceptability criteria for CTT analysis).

**Table 3:** Classical Test Theory Analyses: Properties, Definitions and Acceptability Criteria

Property	Definitions and Acceptability Criteria
Data quality	Data quality refers to the extent to which the scale items are accepted by the participants and, consequently, yield usable responses. Missing data are indicative of a lack of acceptability and/or a lack of applicability of the items from the perspective of the participant. Item-level missing data should be < 10% [58]
Scaling assumptions	Scaling assumptions refer to the extent to which it is legitimate to sum a set of item scores, without weighting or standardisation, to produce a single total score [59, 60]. Summing scale item scores is considered legitimate, when the items: • are approximately parallel (i.e., they measure at the same point on the scale). This criterion is satisfied when items have similar mean scores [61]; • contribute similarly to the variation of the total score (i.e., they have similar variances), otherwise they should be standardized. This criterion is satisfied when items have similar standard deviations [62]; • measure a common underlying construct, as otherwise combining them to produce a single score is not appropriate [63]. This criterion is satisfied when items have adequate corrected item-total correlation (ITC ≥ 0.30) [64]; • contain a similar proportion of information concerning the construct being measured. Otherwise items should be given different weights [61]. This criterion is satisfied when items have similar ITCs [64].
Scale-to-sample targeting	Scale-to-sample targeting refers to the extent to which the range of the construct measured by the scale matches the range of that variable in the study sample. Adequate targeting provides greater confidence in making judgments about the performance of the scale when interpreting results. Poor targeting implies that measurement precision is limited. People with extreme scores represent a sub-sample in which changes within and differences between individuals will be underestimated. Scale scores should span the entire range; floor (proportion of the sample at the minimum score for the scale) and ceiling (proportion of the sample at the maximum score) effects should be low (< 15%) [65]; and skewness, i.e., the third central moment of the distribution capturing its asymmetry, should be between ±1 [66]. There are no published criteria for item-level targeting.
Reliability	Reliability refers to the extent to which scale scores reflect random error. High reliability indicates that scores are associated with little random error, i.e., are consistent. Internal consistency reliability estimates the random error associated with total scores from the intercorrelations among the items [67]. The recommended level for adequate scale internal consistency is Cronbach’s alpha coefficient ≥ 0.80 [67], and item-total correlations > 0.30 [58].

Survey 2 analyses replicated the same analyses on the item-reduced scales obtained from Survey 1 for cross-validation with an independent sample. In addition, construct validity (i.e., convergent and known-group) was evaluated. Convergent validity was assessed by non-parametric correlations with individual items of related measures (i.e., VAS on overall health risk; VAS on addiction risk and the five items on short and long-term consequences of smoking). PRI score differences between respondent groups that were expected to differ based on subject matter considerations (known-group validity) were assessed with t-tests. The group differences examined were: (1) perceived personal versus general risk among current smokers (with perceived personal risk expected to be lower) [39, 40]; (2) current versus never smokers (with perceived risk of smoking expected to be lower for current smokers) [40]; and (3) between smokers with versus without intention to quit (with perceived risk of smoking in smokers intending to quit expected to be higher) [41].

To explore the extent to which the PRI scores were influenced by the position of the assessment object in the sequence, mean scores were calculated by object, sequence and smoking group for all PRI scales, based on RMT logit measures transformed into a 0–100 score. Since the number of sequences was very large (120 possible sequences), the assessment of sequence effects was based on pairwise comparisons of objects using t-tests for independent samples [42].

RMT analyses were performed using RUMM2030 and all other analyses were performed with SPSS (version 21). All statistical tests were conducted at a test-wise alpha level of 5%.

## Results

### Participants

The baseline characteristics of the 2020 and 1640 participants who completed Survey 1 and Survey 2, respectively, are summarized in Table 1. Due to the quota sampling, similar numbers of males and females completed the surveys (46% and 48% of males respectively). Between 61% and 65% of the participants had a high school or higher education and slightly more participants completed Survey 1 in the 46+ years of age group (41% and 36%, respectively). In both surveys, most participants categorized themselves as Caucasians (81% and 80%, respectively). Disposition of participants in Survey 1 and 2 is presented in Table 4.

**Table 4:** Participant Disposition in Surveys 1 and 2

Participant status	Survey 1 *n* (%)	Survey 2 *n* (%)
Accessed the survey	11,914	14,904
Enrolled in the survey	2411	2400
Completed the survey	2020	1640
Dropped out during the survey	391	760
Not enrolled because of inclusion/exclusion criteria violation	2512	2764
Not enrolled because of full quota	3082	4312

### Scale formation and item reduction (Survey 1)

The 34 items assessing Perceived Health Risk demonstrated no disordered thresholds, reasonable coverage of the item thresholds (88%) and good reliability as assessed by the PSI of 0.97 (Table 5). Through a series of three iterations, a total of 16 items were removed from the initial item pool. Although the psychometric red flags were misfit (*n* = 9), and uniform DIF (*n* = 7), the totality of evidence to support the extent to which scales were fit for purpose (i.e., conceptual clarity, contexts of use, intended application and use cross cultural studies) was leveraged in each instance to make the final decisions in relation to item retention. A re-analysis of the reduced 18-item Perceived Health Risk scale (for items see Table 6) revealed that the scale performed appropriately (i.e., no disordered threshold, no DIF for any of the subgroups tested, coverage of 84% of participants, and a PSI of 0.97; Table 5).

**Table 5:** Rasch Measurement Theory –Summary for PRI Health and Addiction Risk Scales in Surveys 1 and 2

Proposed Scale (# items)	% coverage item threshold distribution	% items with fit residual > | 2.5 |^a^	% items with p (χ^2^) < 0.05 ^b^	% items with disordered thresholds	% pairs of item residual correlations > 0.30	% pairs of item residual correlations > mean + 0.30^c^	% items with p (DIF) < 0.05^b^	PSI
Survey 1 Long Form Scales								
Health Risk (34)	88	94	21	0	16/595	24/595	50	0.97
Addiction Risk (11)	80	82	18	0	3/49	4/49	9	0.94
Survey 1 Reduced Scales								
Health Risk (18)	84	61	0	0	0/153	13/153	0	0.97
Addiction Risk (7)	75	86	0	0	0/18	2/18	0	0.93
Survey 2 Reduced Scales								
Health Risk (18)	87	72	0	0	0/153	8/153	0	0.97
Addiction Risk (7)	78	86	0	0	0/18	1/18	0	0.94

*PSI* person separation index, *χ*^*2*^ Chi-square, *DIF* differential item functioning

^a^The high percentages were expected given the large sample size but are still informative when some items are much worse fitting relative to others

^b^In the statistical assessment the actual n was adjusted to 500 in order to mitigate excessive power and for parallel fit assessment based on a sample size of 500, which is deemed appropriate for the present psychometric analysis

^c^The critical values for residual correlations were 0.268 and 0.188, respectively, for Survey 1 Long Form Scales; 0.146 and 0.058, respectively, for Survey 1 Reduced Scale: and 0.169 and 0.057, respectively, for Survey 2 Reduced Scales

**Table 6:** PRI Health and Addiction Risk Items

Domain, item (abbreviated)^a^	Item location	Standard error	χ^2^ (df = 9)	*p* (χ^2^)^b^
PRI Perceived Health Risk				
Cough lasting for days	0.150	0.021	4.612	0.867
Gum health	0.035	0.022	2.275	0.986
Lung cancer	− 0.477	0.021	7.998	0.534
Wheezing	−0.193	0.021	1.421	0.998
Mouth throat cancer	−0.058	0.022	0.931	1.000
Aging faster	−0.015	0.021	0.445	1.000
Minor illnesses	0.176	0.022	1.968	0.992
Respiratory infection	−0.051	0.022	5.752	0.764
Serious illness	0.049	0.022	4.425	0.881
Reduced stamina	0.135	0.022	2.138	0.989
Emphysema	−0.132	0.021	3.447	0.944
Cough in the morning	0.045	0.021	2.879	0.969
Sense of taste	−0.288	0.022	3.543	0.939
Heart disease	−0.147	0.021	0.817	1.000
Earlier death	0.426	0.022	5.717	0.768
Sores mouth throat	0.319	0.022	4.140	0.902
Unfit	0.001	0.022	0.824	1.000
Other cancer	0.150	0.021	4.612	0.867
PRI Perceived Addiction Risk				
Being unable quit	0.428	0.028	6.203	0.719
Feeling addicted	−0.133	0.025	6.343	0.705
To feel better	0.311	0.026	2.750	0.973
Feeling like have to smoke	0.105	0.026	4.742	0.856
Cannot stop	0.230	0.028	3.665	0.932
Feeling unable quit	0.097	0.028	2.853	0.970
Anxiety situation people smoke	−1.038	0.054	10.612	0.303

^a^Full item wording available through MAPI Research Trust

^b^*p* values based on a random sample of *n* = 500

Psychometric performance based on CTT methods was also strong: skewness of 0.05; Cronbach’s alpha of 0.99; and corrected Item-Total Correlations ranging from 0.89 to 0.93 (Table 7). The percentage of missing data was 0.1% at most at the item-level, demonstrating high acceptability of the PRI. The proportion of “don’t know” responses was between 11% and 15%. While “don’t know” responses were valuable qualitative information, they had to be treated as missing data in the psychometric analysis. However, the observed proportion of “don’t know” responses had no adverse consequences for parameter estimation and scale evaluation, given the large number of responses in total.

**Table 7:** Classical Test Theory –Summary for PRI Health and Addiction Risk Scales in Surveys 1 and 2

Proposed Scale (# items)	Range don’t know responses (%)	Min-Max Sum score	Mean Sum score (SD)	Range CITC	Ceiling/ Floor (%)	Skewness	Cronbach’s alpha	Mean IIC	Range IIC
Survey 1									
Health Risk (18)	11–15	18–90	54.4 (22.32)	0.89–0.93	7/10	0.05	0.99	0.83	0.76–0.90
Addiction Risk (7)	8–12	6–30	20.7 (7.50)	0.90–0.93	8/20	−0.41	0.98	0.87	0.82–0.91
Survey 2									
Health Risk (18)	12–14	18–90	56.1 (20.46)	0.88–0.92	5/10	0.02	0.99	0.81	0.75–0.89
Addiction Risk (7)	8–13	6–30	20.6 (7.09)	0.92–0.95	6/18	−0.32	0.98	0.89	0.85–0.93

*SD* standard deviation, *CITC* corrected item-total correlation, *IIC* inter-item correlation

The 11 items assessing Perceived Addiction Risk showed no disordered item thresholds, reasonable coverage of the item thresholds (80%) and good reliability with a PSI of 0.94 (Table 5). Three items showed misfit and one item uniform DIF. Once again, we leveraged all the available evidence to decide on item retention. A re-analysis of the reduced 7-item Perceived Addiction Risk scale (for items see Table 6) revealed that the scale performed appropriately (Table 5). Among the seven items, three are applicable for all objects but for Cessation. One item (*feeling anxiety when in a situation where people smoke*) was retained for administration only for Cessation (4-item scale for Cessation and 6-item scale for all other tobacco and nicotine-containing products). A re-analysis of the reduced 7-item Perceived Addiction Risk scale revealed that the scale performed appropriately: No disordered thresholds, no DIF for any of the subgroups tested, coverage of 75% and a PSI of 0.93 (Table 5). Psychometric performance based on CTT methods was also strong: Skewness of − 0.41; Cronbach’s alpha of 0.98; Corrected Item-Total Correlations ranging from 0.90 to 0.93 (Table 7). The item-level missing data percentages were at 0.1% at most. At the item level, the proportion of “don’t know” responses was between 8% and 12%.

For both the Perceived Health and Addiction Risk scales, the personal versus general risk versions performed equivalently from a psychometric point of view (i.e., no DIF).

### Psychometric cross-validation (Survey 2)

The analysis of the Survey 2 18-item Perceived Health Risk scale data revealed that the scale performed appropriately: no disordered thresholds; no DIF; 87% of coverage of participants; and a PSI of 0.97 (see Table 5 for summary statistics and Table 6 for item statistics). Psychometric performance based on CTT methods was also strong: skewness 0.02; Cronbach’s alpha of 0.99; and Corrected Item-Total Correlations ranging from 0.88 to 0.92 (Table 7).

At the item-level, the percentage of missing data was 0.1% at most, confirming very high acceptability of the PRI. Among completers, 99% of the study participants provided responses to all items, including the “don’t know” option, the latter being treated as missing data in the psychometric analysis. At the item level, the proportion of “don’t know” responses was in the range of 12% and 14%. The item thresholds ranged between − 4.5 and + 4.0 providing for a broad area where the scale was effective allowing for precise and interpretable measurement.

The 7-item Perceived Addiction Risk scale showed no disordered item thresholds, reasonable coverage of the category thresholds (78%) and good reliability with a PSI of 0.94 (see Table 5 for summary statistics and Table 6 for item statistics). Psychometric performance based on CTT methods was also strong: Skewness of − 0.32; Cronbach’s alpha of 0.98; Corrected Item-Total Correlations ranging from 0.92 to 0.95 (Table 7). As for the Perceived Health Risk Scale, the item-level missing data percentages were at 0.1% at most. At the item level, the proportion of “don’t know” responses was between 8% and 13%. The item thresholds of the Perceived Addiction Risk scale ranged between − 5.4 and + 5.3 providing for a broad area where the scale was effective allowing for precise and interpretable measurement.

### Construct validity (Survey 2)

For the assessment objects CC, THS 2.2, E-cigarettes and NRT, all correlations between the VAS scores and PRI measures for both Perceived Health Risk and Perceived Addiction Risk were in the range of 0.52 to 0.68 across both types of risk (i.e., personal and general; Table 8). Assuming a reliability of the VAS of 0.6 and applying the Spearman Brown formula [43] for disattenuation imply correlations in the order of 0.68 and 0.89.

**Table 8:** Convergent Validity of PRI Scales with VAS Scores (Survey 2)

Scale	CC r_s_ (*n*)	THS 2.2 r_s_ (*n*)	E-CIG r_s_ (*n*)	NRT r_s_ (*n*)
PRI-P vs. VAS Health Risk	0.58 (765)	0.65 (651)	0.65 (717)	0.54 (550)
PRI-P vs. VAS Addiction Risk	0.56 (767)	0.67 (704)	0.68 (708)	0.57 (534)
PRI-G vs. VAS Health Risk	0.52 (775)	0.61 (711)	0.62 (724)	0.52 (713)
PRI-G vs. VAS Addiction Risk	0.54 (771)	0.59 (702)	0.61 (714)	0.52 (704)

*CC* Conventional cigarettes, *E-CIG* Electronic cigarettes, *n* number of study participants with both measurements, *NRT* Nicotine Replacement Therapy, *PRI-p* Perceived Risk Instrument-Personal Risk, *PRI-G* Perceived Risk Instrument-General Risk, *r*_*s*_ Spearman rank correlation coefficient, *THS* Tobacco Heating System, *VAS* Visual Analog Scale

Correlations of the 18-item Perceived Health Risk measure with all five items on short- and long-term consequences of smoking were all in the expected direction for both personal and general risk (Table 9). Correlations were mostly weak to moderate, regardless of smoking status and type of risk, with absolute values ranging from 0.10 to 0.40 for personal risk and from 0.20 to 0.46 for general risk. The size of these correlations was not expected to be very high given the specific content of the individual items of the short- and long-term consequences of smoking questionnaire. Importantly, correlations were of similar magnitude across items focusing on short-term (first three items) or long-term consequences of smoking CC (last two items). This provides strong evidence that the 18-item Perceived Health Risk scale is balanced in terms of short- and long-term risks.

**Table 9:** Convergent Validity of PRI 18-Item Health Risk Scale (CC) with Items from the Short- and Long-Term Smoking Risks Questionnaire (Spearman Correlation Coefficients, Survey 2)

	PRI-P Health Risk Scale					PRI-G Health Risk Scale				
Short and Long-Term Risk Questionnaire	All (*n* = 773)	NS (*n* = 184)	FS (*n* = 192)	CS IQ (*n* = 203)	CS NIQ (*n* = 194)	All(*n* = 778)	NS(*n* = 192)	FS (*n* = 196)	CS IQ(*n* = 197)	CS NIQ(*n* = 193)
Item 1	−0.35	− 0.26	−0.40	− 0.21	− 0.21	−0.30	− 0.29	−0.29	− 0.20	−0.33
Item 2	0.33	0.34	0.28	0.24	0.35	0.39	0.26	0.45	0.31	0.45
Item 3	−0.28	−0.27	−0.34	− 0.14	−0.14	−0.29	− 0.26	−0.24	− 0.23	−0.25
Item 4	−0.28	−0.30	− 0.37	−0.10	− 0.13	− 0.28	−0.27	− 0.29	−0.24	− 0.23
Item 5	0.30	0.18	0.18	0.28	0.37	0.41	0.29	0.39	0.36	0.46

*CS IQ* current smokers with intention to quit, *CS NIQ* current smokers with no intention to quit, *FS* former smokers, *NS* never smokers, *n* number of study participants with both measurements, *PRI-P* Perceived Risk Instrument-Personal Risk, *PRI-G* Perceived Risk Instrument-General Risk

Item 1: There is really no risk at all for the first two years

Item 2: Every single cigarette smoked causes a little bit of harm

Item 3: Although smoking may eventually harm this person’s health, the very next single cigarette he or she smokes will probably not cause any harm

Item 4: Harmful effects of smoking rarely occur until a person has smoked steadily for many years

Item 5: Smoking at the daily rate of one package of cigarettes each day will eventually harm this person’s health

Descriptive statistics of the PRI scales by object (Table 10) showed that the perceived risk of CC was always the highest for both Perceived Health Risk and for Perceived Addiction Risk. This was true for personal and general risk. The risk of THS 2.2 was uniformly considered second-highest after CC. E-Cigarettes were perceived to be less risky compared to THS 2.2. The perceived risks of NRT and Cessation generally marked the lower end. Since the risks associated with NRT referred to the risk of using NRT for a certain period of time in the future, while Cessation meant the perception of incurred risks of smoking CC in the past, this could explain that NRT was perceived as more risky than Cessation. It might seem obvious to compare the levels of observed perceived risk with actual objective risk as another way of assessing convergent validity of the PRI. Indeed, the perceived risk of ongoing use of CC was clearly higher than Cessation or using NRT, which was in line with what one would have expected. However, the evaluation of objective risk of E-cigarettes is still a matter of ongoing research and no final assessment has been made yet. Even less is known about the objective risk of THS 2.2. Thus, the potential to compare perceived risks and objective risk is limited. In fact, the lack of objective evidence of risks associated with using THS 2.2 was one of the main reasons to develop the PRI.

**Table 10:** PRI Health and Addiction Object Means

Instrument: Type of Risk Domain	Object	Rasch-Based (logits)
		Mean (SD)
PRI-P: Personal Perceived Health Risk	CC (*n* = 773)	2.12 (3.19)
	THS 2.2 (*n* = 718)	0.51 (3.17)
	E-CIG (*n* = 726)	−0.15 (3.36)
	NRT (*n* = 556)	−1.47 (3.15)
	CESS (*n* = 586)	−0.69 (2.86)
PRI-P: Personal Perceived Addiction Risk	CC (*n* = 770)	2.91 (3.51)
	THS 2.2 (*n* = 706)	1.23 (3.66)
	E-CIG (*n* = 712)	0.61 (3.88)
	NRT (*n* = 537)	−0.30 (3.62)
	CESS, towards CC (*n* = 583)	−0.89 (3.60)
PRI-G: General Perceived Health Risk	CC (*n* = 778)	2.51 (2.88)
	THS 2.2 (*n* = 716)	0.63 (2.97)
	E-CIG (*n* = 728)	−0.17 (3.06)
	NRT (*n* = 718)	−0.70 (3.12)
	CESS (*n* = 767)	0.07 (2.83)
PRI-G: General Perceived Addiction Risk	CC (*n* = 773)	3.73 (3.06)
	THS 2.2 (*n* = 703)	1.69 (3.46)
	E-CIG (*n* = 715)	0.75 (3.40)
	NRT (*n* = 705)	0.30 (3.29)
	CESS, towards CC (*n* = 753)	−0.04 (3.32)

*CC* Conventional cigarettes, *CESS* Cessation, *E-CIG* Electronic cigarettes,* NRT* Nicotine Replacement Therapy, *PRI* Perceived Risk Instrument, *SD* standard deviation, *THS 2.2* Tobacco Heating System 2.2

With respects to known-group validity, all mean differences were in the expected direction. In terms of the effect sizes (Cohen’s d), differences between smokers and never smokers were more pronounced than differences between personal and general risk among current smokers (Table 11). Regarding the differences between current smokers with and without intention to quit, known-group validity was confirmed as well by the perceived risk being higher for smokers with quitting intention.

**Table 11:** Known-Group Validity: Comparison of Perceived Health Risk Score for CC between Different Groups (Survey 2)

Instrument	Smoking Status Group	*n*	Mean (logits)	SD	t (df)	*p*-value	Cohen’s d
Differences between personal and general risk							
PRI-P	CS (all)	397	1.26	2.88	2.50 (785)	0.013	0.18
PRI-G	CS (all)	390	1.77	2.88			
PRI-P	CS NIQ	194	0.93	2.96	1.21 (385)	0.227	–
PRI-G	CS NIQ	193	1.29	2.93			
PRI-P	CS IQ	203	1.58	2.76	2.42 (398)	0.016	0.24
PRI-G	CS IQ	197	2.25	2.76			
Differences between current smokers and never smokers							
PRI-P	CS (all)	397	1.26	2.88	6.28 (579)	<.001	0.53
	NS	184	3.05	3.80			
	CS NIQ	194	0.93	2.96	6.08 (376)	<.001	0.62
	NS	184	3.05	3.80			
	CS IQ	203	1.58	2.76	4.39 (385)	<.001	0.44
	NS	184	3.05	3.80			
PRI-G	CS (all)	390	1.77	2.88	7.53 (580)	<.001	0.68
	NS	192	3.65	2.69			
	CS NIQ	193	1.29	2.93	8.22 (383)	<.001	0.84
	NS	192	3.65	2.69			
	CS IQ	197	2.25	2.76	5.06 (387)	<.001	0.51
	NS	192	3.65	2.69			
Differences between CS IQ and CS NIQ							
PRI-P	CS IQ	203	1.58	2.76	2.28 (395)	0.023	0.23
	CS NIQ	194	0.93	2.96			
PRI-G	CS IQ	197	2.25	2.76	3.33 (388)	0.001	0.34
	CS NIQ	193	1.29	2.93			

*CS IQ* current smokers with intention to quit, *CS NIQ* current smokers with no intention to quit, *FS* former smokers, *NS* never smokers, *PRI-P* Perceived Risk Instrument-Personal Risk, *PRI-G* Perceived Risk Instrument-General Risk, *SD* standard deviation. Cohen’s d indicated for *p*-values < 0.05

### Carry-over effects (Survey 2)

For the assessment objects CC, THS 2.2 and E-cigarettes no differences were detected between measures of Perceived Health Risk when the product was presented first versus second or later (Table 12). However, for Cessation, both personal and general Perceived Health Risk were higher when Cessation was presented as the first assessment object compared to it being presented after any other assessment. For NRT, a similar effect was found for perceived general risk, with the level of perceived risk being higher when NRT was assessed first.

**Table 12:** Assessment of Carry-Over Effects (Perceived Health Risk Scale Survey 2)

Sequence	*n*	Mean (logit)	SD	t (df)	*p*-value	Cohen’s d
PRI-P						
CC first CC subsequently	159 614	2.08 2.13	2.98 3.24	0.18 (771)	0.860	–
THS 2.2 first THS 2.2 subsequently	149 569	0.62 0.48	3.19 3.17	−0.45 (716)	0.650	–
E-CIG first	142	−0.25	3.42	0.39 (724)	0.696	–
E-CIG subsequently	584	−0.12	3.34			
NRT first	110	−1.35	2.85	−0.42 (554)	0.672	–
NRT subsequently	446	−1.49	3.22			
CESS first	115	−0.05	2.52	−2.66 (584)	0.008	0.29
CESS subsequently	471	−0.84	2.91			
PRI-G						
CC first	162	2.89	2.75	−1.89 (776)	0.060	–
CC subsequently	616	2.41	2.91			
THS 2.2 first	149	0.50	2.97	0.62 (714)	0.537	–
THS 2.2 subsequently	567	0.66	2.97			
E-CIG first	143	−0.09	3.21	−0.35 (726)	0.723	–
E-CIG subsequently	585	−0.19	3.03			
NRT first	140	−0.21	2.85	−2.10 (716)	0.037	0.20
NRT subsequently	578	−0.82	3.17			
CESS first	156	0.95	2.76	−4.41 (765)	< 0.001	0.40
CESS subsequently	611	−0.15	2.80			

*CC* Conventional cigarettes, *CESS* Cessation, *E-CIG* Electronic cigarette, *NRT* Nicotine Replacement Therapy, *PRI-P* Perceived Risk Instrument-Personal Risk, *PRI-G* Perceived Risk Instrument-General Risk, *THS 2.2* Tobacco Heating System 2.2. Cohen’s d indicated for *p*-values < 0.05

## Discussion

The psychometric performance of the PRI was strong across both RMT and CTT analyses, supporting the conclusion that the 18-item Perceived Health Risk scale and the 7-item Perceived Addiction Risk scale are reliable and psychometrically valid. Construct validity evaluations of both scales met expectations through inter-scale correlations and findings from known-group comparisons. At the same time, the assessment of convergent validity was limited due to the absence of an undisputed gold standard measure for perceived risk assessment. Specifically, no definitive assessment of objective risks of products, such as E-Cigarettes or THS 2.2, has been made that would allow to compare objective and perceived risks. The PRI scale measures were correlated with single items (VAS or items from the short- and long-term consequences of smoking questionnaire), resulting overall in moderate convergent validity, mostly due to the lack of reliability of single items compared to PRI scale measures.

To enable appropriate use of the PRI, the final outcome of the present study was the development of a calibrated scoring table (available through MAPI Research Trust), based on weighted likelihood estimation (WLE) [44]. Given the participant raw scores and item parameters, the calibration was done with the restricted Rasch model for polytomous responses [45, 46]. For complete data, the resulting conversion table transfers sum scores to logit measures, which are mapped to a 0–100 scale for convenience. The conversion is a simple linear transformation that changes the logit mean of 0 to 50 and converts the most extreme measures to 0 and 100, respectively.

The application of the Rasch model for measurement [45, 46] implied that item discrimination was supposed to be the same across all items in a scale. While this property of the model provides for invariance in the parameters of the model as an advantageous property facilitating generalizability, it undoubtedly represents a restriction to the data. More general item response theory (IRT) models, such as the Generalized Partial Credit model [47], account for different item discrimination by estimating additional parameters. However, in the case of the PRI, the assumption of equal discrimination was empirically supported. Therefore, estimating discrimination parameters would have run contrary to the general scientific principle of parsimony and would not have significantly improved the fit of the model to the data.

The relatively high item-intercorrelations (between 0.75 and 0.89 for Perceived Health Risk in Survey 2; Table 7) could be of concern as a potential indication of item redundancy. However, no specific pair of items stood out with respect to the item-intercorrelation. Rather, the high correlations were a result of consistency in the response patterns and high measurement precision. What is more, redundancy was a key criterion in the data analysis by examining residual correlations. In the item reduction phase, any potential duplication of content was thoroughly considered ensuring that the final scales lack any redundancy.

There are four key strengths in this instrument development program. First, the content validity of the new instrument (that is the scale scores represent the concepts of interest, and the instructions and item content are appropriate, comprehensive and understandable to the target population) was evidenced by information gathered from literature review, focus groups, expert opinions, cognitive debriefing interviews, and pilot field testing.

Second, the design of the quantitative studies included a broad range of subpopulations in the US in terms of smoking status, considering current and past smoking behaviour as well as intentions to quit smoking cigarettes. The diversity of subpopulations provided a broad frame of reference for which the validity of the PRI could be demonstrated. In addition, the sample design provided an approximately equal representation of all four smoking status groups, ensuring adequate psychometric analysis for all groups. Within each smoking status group, additional stratification allowed for the assessment of measurement equivalence across age groups, sex and levels of education. The psychometric cross-validation with an independent sample and the large total sample size support a robust psychometric quality of the items.

Third, the fit of the data to the unidimensional measurement model and the lack of DIF by assessment object demonstrate that the items for each scale worked as a set, representing manifestations of unidimensional perceived health and addiction risks, respectively, for a diversity of products, i.e., combustible cigarettes, heat-not-burn product, e-cigarettes, nicotine replacement therapy products as well as Cessation. Therefore, the instrument development provides a solid foundation for the scales to be used with other products (e.g., different potential RRPs). Nevertheless, for application of the PRI to products substantially different to those assessed here, such as smokeless tobacco, reinvestigating the validity of the scales is advisable. In particular, the comparability of the perceived risk measures with those related to products considered in the scale development project should be assessed at the item level by DIF analyses.

Fourth, the instrument development accounted for two types of risk perceptions: personal risk (risk to the individual respondent) and general risk (risk to users of the products in general). Both personal (PRI-P) and general (PRI-G) versions of the instrument performed equally well from a psychometric point of view, implying that either of the two could be used in future studies, depending on the design and objective.

There are also some limitations to our study. First, web panels are not fully representative of the US population. In particular, a bias towards higher education is a typical and widespread phenomenon in panel-based online surveys [48]. In order to mitigate this limitation, education was included as a sampling quota. The relative simplicity of the items, their high comprehensibility as demonstrated in the CDIs and the very low rates of non-completers dropping out of the survey prematurely suggest the suitability of the PRI for a broad range of educational levels. This conclusion was also supported by evidence from the psychometric analyses, with DIF analyses confirming that the scales work equivalently for participants with higher and lower education.

Second, as the study was administered as a web survey, all psychometric findings are in principle confined to this mode of administration. As a suggestion for future research, the administration of the PRI as a paper-and-pencil questionnaire or as a telephone interview should involve a cross-method comparison of the psychometric properties. The likelihood of the validity of the PRI to be maintained when administered in modes other than online, particularly through paper-and-pencil, is deemed high, as the instruments proved very stable in terms of diverse subpopulations (e.g., based on smoking status) and objects (products, behaviours). The simplicity of the items themselves also contributes to high comprehensibility of the PRI, as demonstrated in the qualitative phase (cognitive debriefing interviews), which indeed included the presentation of the instruments on paper.

Third, the assessment of perceived health risks concerning different types of tobacco and nicotine-containing products was not completely free of carry-over effects. In principle, fit of the data to the Rasch model supports specifically objective measurement and, thus, invariance. However, specific objectivity only applies within a frame of reference, for which invariance of comparisons has been empirically demonstrated [49]. The analysis of repeated measurements of perceived risks provided evidence that the study design may jeopardize invariance and, therefore, comparability of measures. The assessment of one type of tobacco and nicotine-containing product may have a priming effect on the subsequent assessment of another product. Studies applying the PRI in a repeated measurement design should thus take the potential of carry-over effects into account, particularly if perceived risks of Cessation and of NRTs are to be assessed. Our findings suggest that these effects may best be accommodated by a fixed order of objects presented to the participants. The best-known product should be presented first, to set a meaningful reference point. Thereafter, tobacco products should be presented by decreasing familiarity. Based on the principle of moving from use of products to their non-use, objects related to quitting smoking should be presented last, with Cessation (not involving any use of NRT) to be presented as the very last object.

Finally, a possible concern when applying the PRI to multiple objects in a repeated measurement design could be response burden. However, the structural simplicity of the PRI consisting of items that are brief statements allows for a straightforward and fast completion (less than 5 min per object). We did consider a shorter 9-item version of the Perceived Health Risk scale. In terms of traditional reliability, the short version would only be slightly less reliable. The standard error of measurement for an individual respondent, though, would increase by up to about 80%, depending on the level of perceived risk. Given the predominant role of perceived health risk from a respondent’s perspective, we therefore recommend the application of the full 18-item scale.

## Conclusions

By quantifying perceived tobacco and nicotine-containing product risks, the PRI fills an important methodological gap and may be used in clinical and population-based studies. Based on the structured development process and the amount of validation data, the PRI can be a valuable self-report instrument that provides a scientifically rigorous method to quantify the perceived risks of tobacco and nicotine-containing products and related behaviors. With increasing numbers of researchers incorporating the PRI into their studies, we envision a rapidly expanding knowledge-base, informing further interpretation of risk perception data comparing a large spectrum of tobacco and nicotine products, so that the health and public policy communities can make more informed decisions on the potential public health impact of MRTPs. Such data will provide meaningful information on: (1) the effects of risk perception on tobacco and nicotine-containing product use behavior among current tobacco users; (2) the effects on product use initiation among non-users; and (3) the effects of risk communication on consumer understanding and perception.

## Abbreviations

CC: Conventional cigarette
CDI: Cognitive debriefing interview
CTT: Classical test theory
DIF: Differential item functioning
FDA: Food and Drug Administration
ICF: Informed consent form
MRTP: Modified risk tobacco product
NRT: Nicotine replacement therapy
PRI: Perceived Risk Instrument
PSI: Person separation index
RMT: Rasch measurement theory
RRP: Reduced risk products
THS 2.2: Tobacco Heating System 2.2
VAS: Visual analog scale
WLE: Weighted likelihood estimation

## Footnotes

1 The three experts involved in the literature review have strong expertise in public health and quality of life, consumer risk perception, qualitative and quantitative research in scale development. They currently hold, or previously held, positions at governmental regulatory bodies, universities or contract research organisations.

2 The four experts involved at this stage were subject matter key opinion leaders (KOLs) in fields of nicotine and other addictions, motivational aspects of consumer perception, in epidemiologic study design, data management, and evaluation, measurement of clinical concepts, evidence-based medicine, and statistical analysis in health. All experts hold leading positions at universities or organisations in health care in the USA or Canada. The KOLs have a long record of publications in the healthcare sector and have contributed to governmental programs at treating various kinds of addictions in public health. None of the four experts was involved in the previous literature review.

3 Reduced Risk Products (“RRPs”) is the term used by Philip Morris Products S.A. to refer to products with the potential to reduce individual risk and population harm in comparison to smoking cigarettes. More details are available on www.pmiscience.com.
